# Hox gene expression determines cell fate of adult periosteal stem/progenitor cells

**DOI:** 10.1038/s41598-019-41639-7

**Published:** 2019-03-25

**Authors:** Vivian Bradaschia-Correa, Kevin Leclerc, Anne M. Josephson, Sooyeon Lee, Laura Palma, Hannah P. Litwa, Shane S. Neibart, Jason C. Huo, Philipp Leucht

**Affiliations:** 1 Department of Orthopaedic Surgery, New York University School of Medicine, New York, NY USA; 2 Department of Cell Biology, New York University School of Medicine, New York, NY USA

## Abstract

*Hox* genes are evolutionarily conserved transcription factors that during embryonic development function as master regulators of positional identity. In postnatal life, the function of *Hox* proteins is less clear: *Hox* genes are expressed during tissue repair, but in this context their function(s) are largely unknown. Here we show that *Hox* genes are expressed in periosteal stem/progenitor cells in a distribution similar to that during embryonic development. Using unbiased sequencing, we established that periosteal stem/progenitor cells from distinct anatomic sites within the skeleton significantly differ in their transcriptome, and that *Hox* expression status best defines these differences. Lastly, we provide evidence that *Hox* gene expression is one potential mechanism that maintains periosteal stem/progenitor cells in a more primitive, tripotent state, while suppression of *Hox* genes leads to fate changes with loss of tripotency. Together, our data describe an adult role of *Hox* genes other than positional identity, and the modulatory role of *Hox* genes in fate decisions may offer potential druggable targets for the treatment of fractures, non-unions and bone defects.

## Introduction

During embryonic development, homeobox (*Hox*) genes establish the cranial-caudal ‘Bauplan’ of the body (reviewed in^1^). In the caudal region, *Hox* genes are expressed in a nested pattern that terminates in the cranial region in the expression of a single *Hox* gene (reviewed in^2^). Anterior to the second branchial arch, from which the mandible and hyoid bones form, skeletal tissues are *Hox*-negative^3^. This *Hox*-positive and *Hox*-negative status of the skeletal elements is maintained into adulthood^4–7^, but why these genes remain transcriptionally active throughout the entire lifespan is not known. In adult animals and humans, the skeleton loses its potential to regenerate missing segments, and therefore a ‘Bauplan’ for restoration is not essential. Yet the continued presence of *Hox* expression in the mature skeleton argues for an alternate function; here, we tested the hypothesis that *Hox* status regulates the fate of periosteal stem/progenitor cells, which are ultimately responsible for healing skeletal injuries.

Periosteal stem/progenitor cells, irrespective of their anatomical origin, are thought to be one cell population, equal in function and character; and thus far, studies have not revealed significant differences in the properties of periosteal stem/progenitor cells from different skeletal elements. If *Hox* genes in fact regulate periosteal stem/progenitor cells function, then this would add another layer of complexity to this sparsely characterized stem/progenitor cell^8^; and our research thus aims at investigating whether the presence or absence of *Hox* expression imparts differential functional information that influences regenerative behavior of the periosteal stem/progenitor cell.

While most musculoskeletal research over the last few decades has focused on bone marrow-derived stromal cells, more recent scientific advances have focused on the periosteal stem/progenitor cell niche. In particular, the periosteal stem/progenitor cell pool demonstrates greater self-renewal, more regenerative potential, and superior *in vitro* proliferative capacity^9^. This heightened interest has resulted in the identification of a unique surface marker profile describing the periosteal stem/progenitor cell^9–11^.

In this study, we establish that *Hox* expression status regulates adult periosteal stem/progenitor cell lineage commitment. We observe a more osteogenic phenotype in *Hox*-negative periosteal stem/progenitor cells, while *Hox*-positive periosteal stem/progenitor cells are more chondrogenic and adipogenic. Gene silencing approaches, using siRNA and antisense oligonucleotides (ASOs) against the long noncoding RNAs *Hotairm1* and *Hottip*, suppress *Hox* expression in *Hox*-positive periosteal stem/progenitor cell populations, and this *Hox*-suppression led to a transcriptional and phenotypic change suggestive of a reversal of lineage commitment. These data demonstrate that *Hox* proteins are developmentally conserved master regulators of stem/progenitor cell fate during wound healing.

## Results

### Embryonic Hox gene signature is maintained in adult skeletal stem cells

**Figure 1:**
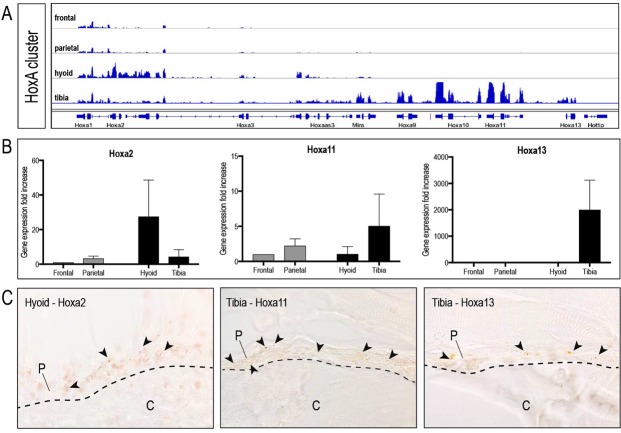
Embryonic *Hox* status of periosteal stem/progenitor cells is preserved into adulthood. (**A**) Transcriptional map depicting normalized FPKM expression values for genes within the HoxA cluster. Note the near absence of *Hox* expression in the frontal and parietal bone, while proximal *Hox* genes are represented in the hyoid sample, and distal *Hox* genes are expressed in the tibia, similar to their embryonic pattern. Expression values from isolated periostea were averaged for each skeletal element (n = 3). (**B**) qPCR validation (mean +/− standard error) of three relevant Hox genes (*Hoxa2, Hoxa11* and *Hoxa13*) identified as differentially expressed by RNA sequencing (n = 3). (**C**) *In situ* hybridization of hyoid and tibial periosteum with *Hoxa2, Hoxa11* and *Hoxa13* RNA antisense probes confirming spatial expression of the respective *Hox* genes within the periosteum (arrowheads)(n = 3). Abbreviations: c, cortical bone; p, periosteum.

“General Purpose” control genes, such as the *Hox* gene cluster, control the body plan of the embryo along the anterior-posterior axis (reviewed in^12^). During this process, patterns of *Hox* gene activity assign each anatomic body part a segmental identity, which culminates in the creation of a complex tissue, organ or organism. While such “Bauplan” is essential during development, it becomes less clear why these control genes would be necessary during adulthood. The most likely function may be found during regeneration of an injured tissue. Here, stem cells, once activated, organize within the regenerate to restore form and function of the injured body part, and it is in this scenario that a body plan gene cluster may provide vital regulatory function. We hypothesized that *Hox* genes continue to function as “general purpose” genes far into adulthood, and in order to test this conserved function of the *Hox* gene cluster, we made use of the skeleton, a contiguous organ, spanning the entire body from cranial to caudal. The skeleton is one of the few adult tissues that regenerates instead of repair/scar^13^ and contains skeletal progenitor cells that are located within distinct anatomic sites of the skeleton, such as the periosteum^10,14–16^. First, we had to confirm that indeed *Hox* gene expression is conserved and present in adulthood. Periosteal stem/progenitor cells were harvested from four anatomic locations^4^, spanning the entire body, and were subjected to transcriptional profiling. RNAseq analysis revealed that embryonically *Hox*-negative periosteal stem/progenitor cells maintained their *Hox*-negative status into adulthood (Fig. 1A), while embryonically *Hox*-positive periosteal stem/progenitor cells continued to express *Hox* genes (Fig. 1A). qRT-PCR analysis confirmed that anterior *Hox* genes continued to be expressed in the hyoid, while posterior Hox genes, such as *Hoxa11* and *Hoxa13*, were expressed in periosteal stem/progenitor cells originating from the tibia (Fig. 1B). These findings were confirmed using *in situ* hybridization, demonstrating expression of the anterior Hox gene, *Hoxa2*, in the cambial layer of the hyoid periosteum, while the posterior Hox gene, *Hoxa11* and *Hoxa13*, were expressed within the tibial periosteum (Fig. 1C).

### Transcriptome analysis reveals difference between Hox-negative and Hox-positive periosteal stem/progenitor cells

**Figure 2:**
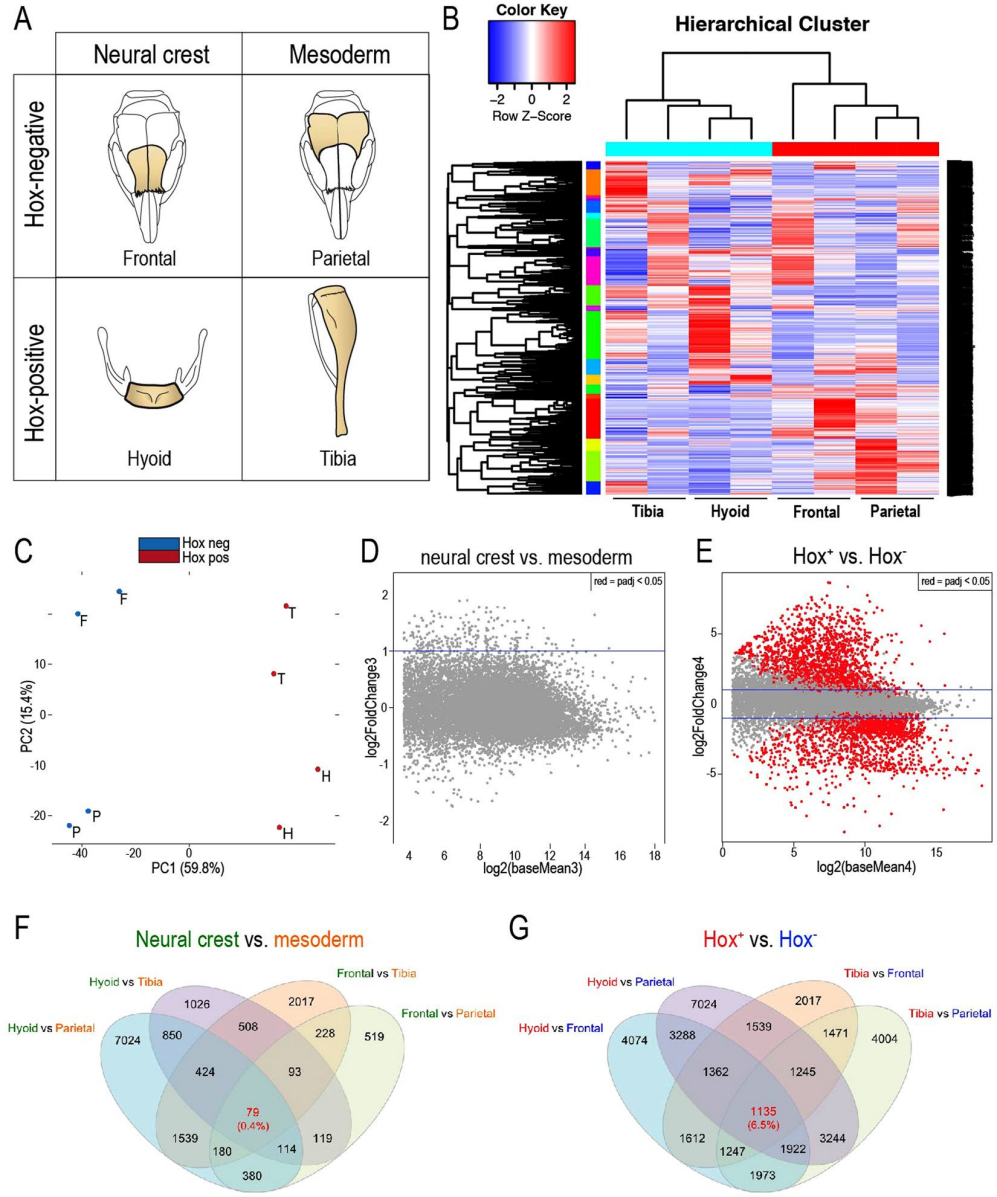
*Hox* status defines SSC identity. (**A**) Comparison of 4 groups, unique in their embryonic origin and *Hox* status, allow for transcriptome comparison with RNAseq. (**B**) Hierarchical clustering analysis of top 1000 most divergent genes. For each gene, we calculated its coefficient of variation (CV) based on its log-transformed FPKM values across all RNAseq samples. The genes were then ranked based on their CV values. The heatmap was generated by hierarchical clustering of the top 1000 genes with the largest CV values. (**C**) Principal component analysis (PCA) of frontal (F), parietal (P), hyoid (H) bone and the tibia (T). (**D**) MA plot comparing differential gene expression between neural crest and mesoderm derived SSCs, and between (**E**) *Hox*-positive and *Hox*-negative SSCs. (**F**,**G)** Integration of both RNAseq and ATACseq data sets reveals genes that were differentially regulated (differential expression by RNAseq and differential open chromatin by ATACseq). Again, Hox^+^ vs. Hox^−^ best described their differences, as 6.5% of the genes were differentially regulated in this comparison (RNAseq: p < 0.05, ATACseq: p < 0.05).

Besides *Hox* gene cluster expression, periosteal stem/progenitor cells present another unique identifying signature: distinctive embryonic origins^4^. Most of the craniofacial skeleton, except for the parietal bone^17^, are derived from the neural crest^18^, while the entire axial and appendicular skeleton are derived from the mesoderm^19^ (Fig. 2A). These different embryonic origins, superimposed with a distinct Hox gene expression pattern, allowed us to define four unique periosteal stem/progenitor cell populations, different in embryonic origin and *Hox* expression (Fig. 2A). If in fact *Hox* gene expression imprints a certain positional identity to these periosteal stem/progenitor cells, then transcriptional profiling using RNAseq should be able to identify which distinct feature (embryonic origin or *Hox* expression) better describes these stem/progenitor cell populations. Hierarchical cluster analysis of the transcriptome of the frontal, parietal, hyoid and tibial periosteal stem/progenitor cells was carried out. Clustering revealed that transcriptional similarities assigned the periosteal cells into two clusters defined by their *Hox* expression status: Periosteal stem/progenitor cells without *Hox* gene expression (frontal and parietal) homogenously clustered together and separately from periosteal stem/progenitor cells with *Hox* gene expression (hyoid and tibia)(Fig. 2B). To further dissect the discrepancies/uniformities between the four stem/progenitor cell origins, we built a principal component analysis (PCA) that plotted the different anatomic origins apart from each other (Fig. 2C). The separation of each cluster confirms that each anatomic site gives rise to a periosteal stem/progenitor cell with a unique transcriptional signature; however, as shown in the hierarchical cluster analysis, ultimately the respective *Hox*-positive and *Hox*-negative periosteal stem/progenitor cells are transcriptionally more similar. MA plots, describing the logarithmic differences between two cell populations, were then built in order to provide a visual representation of the transcriptional similarities/differences either between periosteal stem/progenitor cells from neural crest- (frontal and hyoid) and mesoderm-derived (parietal and tibia) skeletal elements (Fig. 2D) or between periosteal stem/progenitor cells with or without endogenous *Hox* gene expression (Fig. 2E). The MA plot distinguishing periosteal stem/progenitor cells according to their embryonic origin revealed that periosteal stem/progenitor cells (SSCs) from NC-derived and MD-derived bones demonstrated significant differences in FPKM reads of 216 genes (Fig. 2D). However, when plotted as *Hox*-expressing and *Hox*-negative SSCs, 5,390 out of 17,569 genes measured by RNAseq revealed statistically different expression levels (Fig. 2E, red dots), indicating that if there is a functional difference between periosteal stem/progenitor cells from the craniofacial and appendicular skeleton, then this difference can be best described by the cells’ *Hox* gene expression status. To further understand this difference in transcriptional signature, we performed ATACseq to identify genes that were not only differentially expressed but also differ in their chromatin accessibility. Integration of the RNAseq and ATACseq data allowed us to identify genes that are differentially regulated between the comparisons. In line with the RNAseq data, the integrated analysis revealed that only 79 genes were differently expressed in cells from the neural crest versus mesoderm (Fig. 2F). However, in the comparison of *Hox*-positive and *Hox*-negative SSCs, 1135 genes exhibited differential regulation (Fig. 2G), again supporting our hypothesis that the *Hox* gene expression status best differentiates SSCs from distinct anatomic skeletal regions.

### Hox-negative and Hox-positive periosteal stem/progenitor cells respond differently to injury

**Figure 3:**
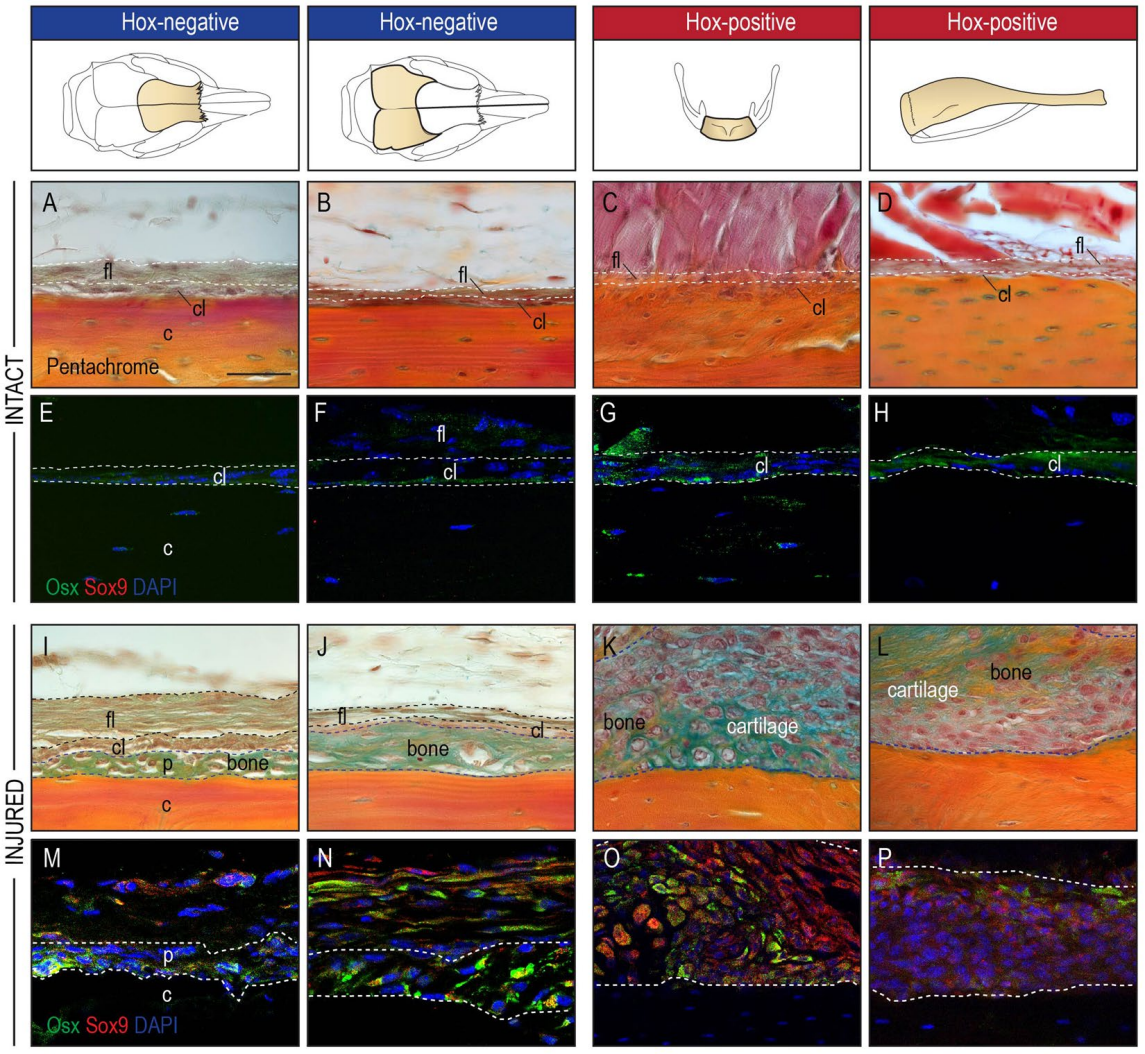
*Hox* status imparts unique regenerative response on periosteal cells. (**A**–**H**) Representative histologic appearance of the periosteum of frontal, parietal, hyoid bone and tibia with characteristic one-cell-layer thick cambial layer (cl) containing *osx*-positive bone-lining cells (green cells in immunofluorescence staining)(**E**–**H**), and a thicker fibrous layer (fl) in the periphery (n = 5). (**I**,**J**) A periosteal scratch injury results in a pure osteogenic response (between dashed line) of the periosteum in the frontal and parietal bone, while (**K**,**L**) periostea from the hyoid and tibia responded with a mixed osteochondrogenic response (*osx* for osteogenesis, *sox9* for chondrogenesis)(n = 5). (**M**,**N**) Immunofluorescence staining of the periosteal injury confirmed the osteogenic response with *Osx*-positive cells within the regenerate (between white dashed lines) and an absence of *Sox9*-expressing cells. (**O**,**P**) In contrast, the periosteal response to injury (between white dashed lines) of the hyoid and tibia exhibited both *Osx*+ and *Sox9*+ expressing cells, in line with a mixed osteochondrogenic healing response. Abbreviations: c, cortical bone; cl, cambial layer; fl, fibrous layer; p, periosteum.

We have previously shown that skeletal stem/progenitor cells reside within the periosteum of the tibia (appendicular skeleton) and the mandible (craniofacial skeleton), and contribute to repair and regeneration of each specific skeletal element after injury^4^. RNAseq data have now revealed that *Hox*-expressing periosteal stem/progenitor cells are transcriptionally different than *Hox*-negative periosteal stem/progenitor cells. We next sought to examine these cells *in vivo* and investigate whether the transcriptional difference results in a morphological change in an uninjured and injured state. Histologic evaluation of uninjured periostea from *Hox*-negative and *Hox*-positive bones revealed an identical anatomic composition with its characteristic two-layer structure (Fig. 3A–H). In response to a scratch injury, *Hox*-negative periostea responded with a pure osteogenic reaction within the layer immediately adjacent to the cortical bone (Fig. 3I,J,M,N). In stark contrast, *Hox*-positive periostea exhibited a mixed response with both cartilaginous and osseous components (Fig. 3K,L,O,P). Immunofluorescence for *Osx* and S*ox9* demonstrated a prevalence of *Osx*-positive cells in the periostea of *Hox*-negative bones after injury, further confirming the more osteogenic phenotype of this periosteum (Fig. 3M,N). In contrast, periostea from *Hox*-positive bones showed a mixture of *Osx*-positive and *Sox9*-positive cells, confirming the histological appearance of a mixed chondrogenic and osteogenic injury response (Fig. 3O,P).

### Hox-positive periostea contain more primitive periosteal stem/progenitor cells

**Figure 4:**
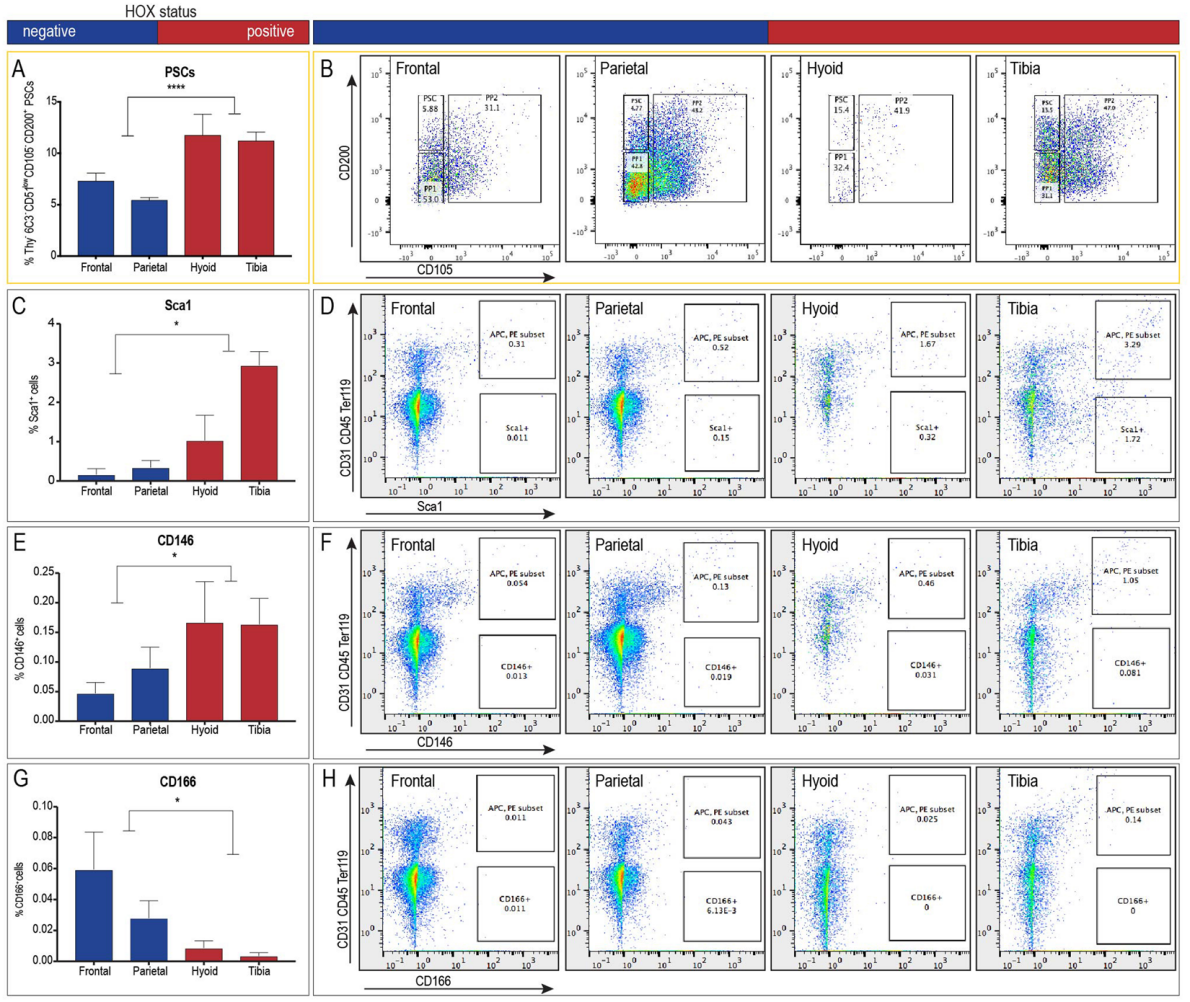
*Hox*-positive periostea contain primitive periosteal stem/progenitor cells. (**A**–**H**) Flow cytometry of *Hox*-negative (frontal and parietal) and *Hox*-positive (tibial and hyoid) periosteal cells (n = 3). (**A**,**B**) Hox-positive skeletal elements contain greater numbers of periosteal stem cells (PSCs), characterized using a gating strategy described by Debnath *et al*.^10^. Negative selection of the endothelial (CD31), lymphoid (CD45), erythrocyte (Ter119), and other committed progenitor (Thy, 6C3, and CD105) compartments revealed the subset of cells that were CD200^+^ PSCs (**B**) Negative selection of the CD31, CD45, Ter119 compartments revealed the subset of cells that were Sca1^+^ (**D**), CD146^+^ (**F**), CD166^+^ (**H**). The left panels demonstrate the proportion of periosteal stem cells (**A**), Sca1^+^ (**C**), CD146^+^ (**E**), and CD166^+^ (**G**) cells as a percentage of total periosteal cells. *p < 0.05, ***p < 0.001. Error bars are SEM.

Besides their transcriptional differences and their unique response to injury, periosteal cell composition demonstrated a clear distinction between *Hox*-positive and *Hox*-negative periostea. *Debnath et al*. recently described a periosteal stem/progenitor cell hierarchy^10^. Using their gating strategy, we observed significantly greater numbers of periosteal stem/progenitor cells in the Hox-positive hyoid and tibia (Fig. 4A,B). In addition, we surveyed other general surface marker combinations that have been used in the past to describe stem/progenitor cells in the skeletal system. FACS analyses revealed that the *Hox*-positive periosteum was enriched in Sca1- and CD146-expressing cells (Fig. 4C–F), while the *Hox*-negative periosteum contained more CD166-positive cell than the *Hox*-positve tissue (Fig. 4G,H). Both Sca1 and CD-146 are associated with the more primitive skeletal/periosteal progenitor cell^20^, while CD166 is labeling cells that have progressed on the lineage tree towards an osteoprogenitor cell^20^. These data support the observation that the *Hox*-positive periosteum is comprised of more primitive cells with both chondrogenic and osteogenic potential, while the *Hox-*negative calvarium is more differentiated and thus less plastic in response to injury.

### Hox gene expression imparts skeletal multi-lineage differentiation potential on postnatal periosteal stem/progenitor cells

**Figure 5:**
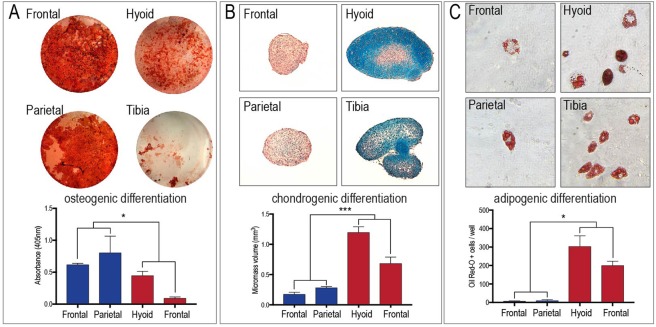
Tri-lineage differentiation potential of *Hox*-positive and *Hox*-negative periosteal cells. Osteogenic (**A**), chondrogenic (**B**) and adipogenic (**C**) differentiation assays of frontal, parietal, hyoid, and tibia periosteal progenitors, as revealed by alizarin red, alcian blue, and oil red O staining, respectively (n = 3). *Hox*-negative periosteal stem/progenitor cells are almost exclusively osteogenic, while *Hox*-positive periosteal stem/progenitor cells exhibit tripotency. *p < 0.05, ***p < 0.001.

We previously demonstrated that craniofacial periosteal stem/progenitor cells exhibit superior osteogenic differentiation compared to appendicular periosteal stem/progenitor cells in an *in vitro* mineralization assay^4^. Having now established that adult periosteal stem/progenitor cells come in two transcriptional flavors, *Hox*-positive and *Hox*-negative, we utilized functional *in vitro* differentiation assays to test whether this previously described transcriptional difference results in a measurable functional difference in osteogenic, chondrogenic and adipogenic differentiation. Periosteal stem/progenitor cells were subjected to tri-lineage differentiation. Osteogenic differentiation resulted in confluent mineralization of the frontal and parietal periosteal stem/progenitor cells assays, while hyoid and tibial periosteal stem/progenitor cells showed significantly less mineralization (Fig. 5A). Chondrogenic differentiation and adipogenic differentiation exhibited the exact opposite differentiation pattern with significantly more chondrogenic and adipogenic differentiation of periosteal stem/progenitor cells derived from the hyoid and tibia (Fig. 5B,C). These differentiation data reveal a striking similarity to the previously shown transcriptional separation of *Hox*-positive and *Hox*-negative periosteal stem/progenitor cells. Here, *Hox*-positive periosteal stem/progenitor cells display a more chondrogenic and adipogenic phenotype, while *Hox*-negative periosteal stem/progenitor cells are more osteogenic.

**Figure 6:**
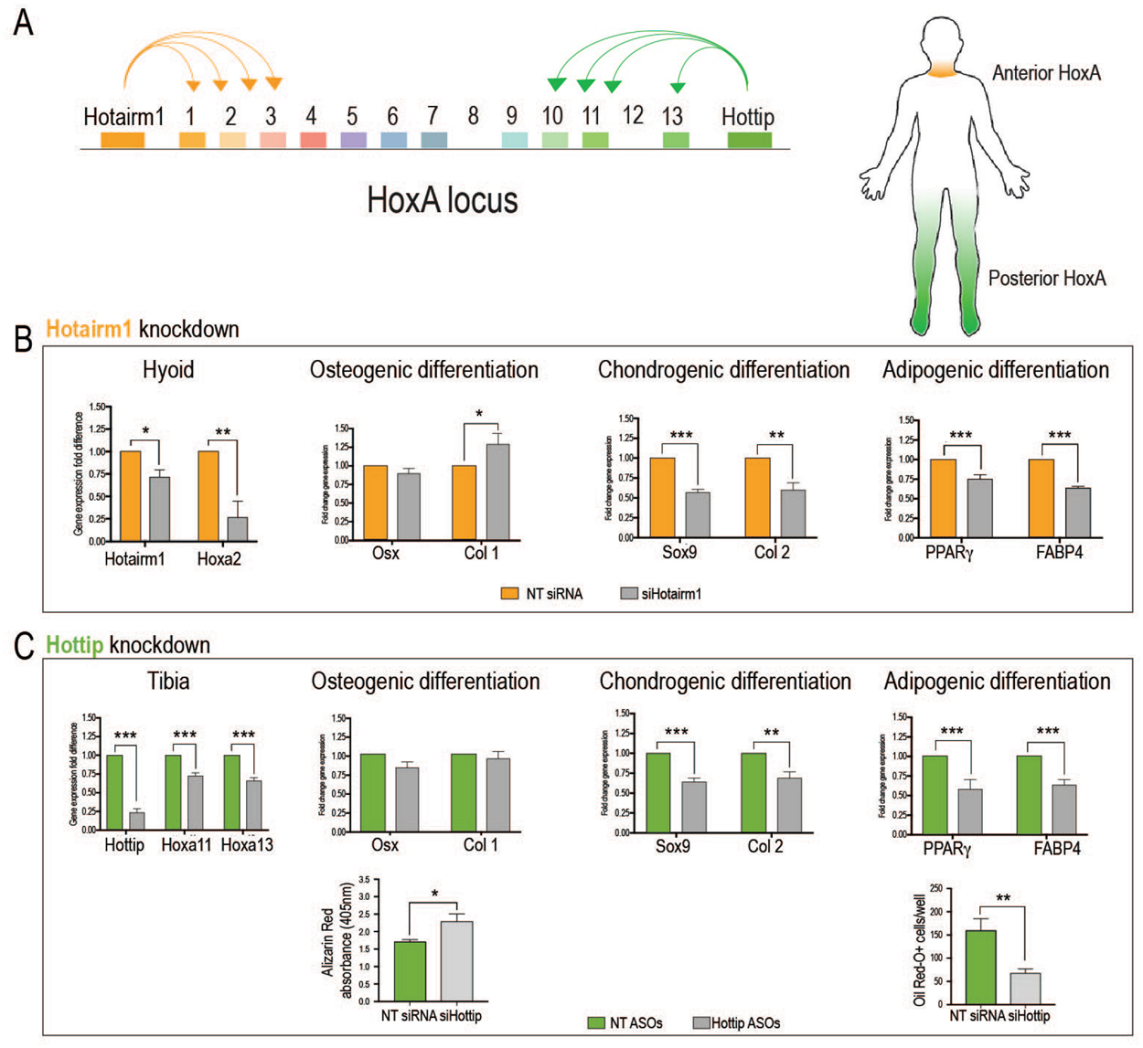
Manipulation of *Hox* gene expression in periosteal stem/progenitor cells leads to cell fate changes. (**A**) Graphical schematic of the mammalian HoxA locus and adjacent lncRNA regulators (left) and the corresponding anatomical regions where anterior and posterior HoxA genes are preferentially expressed (right). (**B**,**C**) Using siRNA and antisense oligonucleotides (ASO) against the lncRNAs *Hotairm1* and *Hottip* to knockdown 5′ and 3′ *Hox* clusters. Knockdown of *Hoxa2* in hyoid periosteal stem/progenitor cells (**B**) and *Hoxa13* and *Hoxa11* in tibial periosteal stem/progenitor cells (**C**) using siRNA against Hotairm1 and ASOs against Hottip resulted in a change in cell fate with increased osteogenic differentiation (*Osx* and *Collagen type I*, hyoid only) and decreased chondrogenic (*Sox9* and *Collagen type II*) and adipogenic differentiation (*Ppar-gamma* and *FabpP4*)(n = 3). (**C**, lower panel) *Hox*-deficient tibial periosteal cells, via *Hottip* knockdown, displayed a greater capacity to differentiate into the osteogenic lineage, as measured by alizarin red absorbance (**C**, lower left panel), and less adipogenic potential, as measures by Oil Red O^+^ cells/well (**C**, lower right panel) when compared with NT control. Abbreviations: NT, non-targeted control. *p < 0.05, **p < 0.01, ***p < 0.001.

In order to test whether cell fate decisions of periosteal stem/progenitor cells during these differentiation assays are *Hox*-dependent, we utilized a knock-down approach targeting lncRNAs that regulate *Hox* expression of hyoid and tibial OPCs (Fig. 6A). *Hotairm1*, a lncRNA located on the noncoding strand and situated at the 3′ end of the HoxA cluster (close to *Hoxa1* and *Hoxa2)*, has been shown to regulate *Hox* expression at this 3′ end (reviewed in^21^). We utilized an siRNA approach to silence the anterior *Hoxa* cluster and then assessed the effect of *Hox* repression on osteogenic, chondrogenic and adipogenic differentiation. siHOTAIRM1 resulted in a significantly lower expression of *Hotairm1* in hyoid periosteal stem/progenitor cells (Fig. 6B). This led to a significant reduction of *Hoxa2* expression in these cells, confirming the successful knockdown (Fig. 6B). If our hypothesis that *Hox* expression leads to decreased osteogenic and increased chondrogenic and adipogenic differentiation, is correct, then we should observe a reversal after *Hox* gene knockdown. Indeed, we detected an increase in osteogenic differentiation of hyoid periosteal stem/progenitor cells after *Hoxa2* knockdown, as shown by a significant increase in *Collagen type 1* expression. In contrast, chondrogenic differentiation, measured by *Sox9* and *Collagen type 2* expression, and adipogenic differentiation, measured by *Ppar-gamma* and *Fabp4* expression, was significantly decreased after *Hoxa2* knockdown (Fig. 6B).

Next, we sought to suppress *Hoxa* cluster expression at the 5′ end, and here antisense oligonucleotides (ASO) were used to knockdown the lncRNA *Hottip*. *Hottip* is expressed in posterior anatomic locations and has been shown to be expressed in a conserved pattern from development to adulthood, where it regulates the expression of the distal 5′ *Hox* cluster^22^. Using an ASO approach, we successfully achieved *Hottip* knockdown in tibial periosteal stem/progenitor cells, and in response, we observed a significant decrease in *Hoxa11* and *Hoxa13* expression levels (Fig. 6C). While our approach was unable to detect a significant difference in osteogenic differentiation on a transcriptional level, there was a significant increase of mineralization detected in a functional osteogenic differentiation assay. *Hoxa11* and *Hoxa13* suppression using *Hottip* ASOs also resulted in a significant reduction of *Sox9* and *Collagen type 2* expression, suggesting a reduction in chondrogenic potential. Finally, *Ppar-gamma* and *Fabp4* expression was significantly decreased after *Hottip* ASO treatment, and the function readout of adipogenesis using oil-red-O staining revealed a decrease in adipogenesis after *Hottip* knockdown (Fig. 6C), confirming our hypothesis that *Hox* gene expression is intimately involved in adult periosteal stem/progenitor cell differentiation.

### RNAseq and ATACseq confirm stemness of Hox-positive periosteal cells

**Figure 7:**
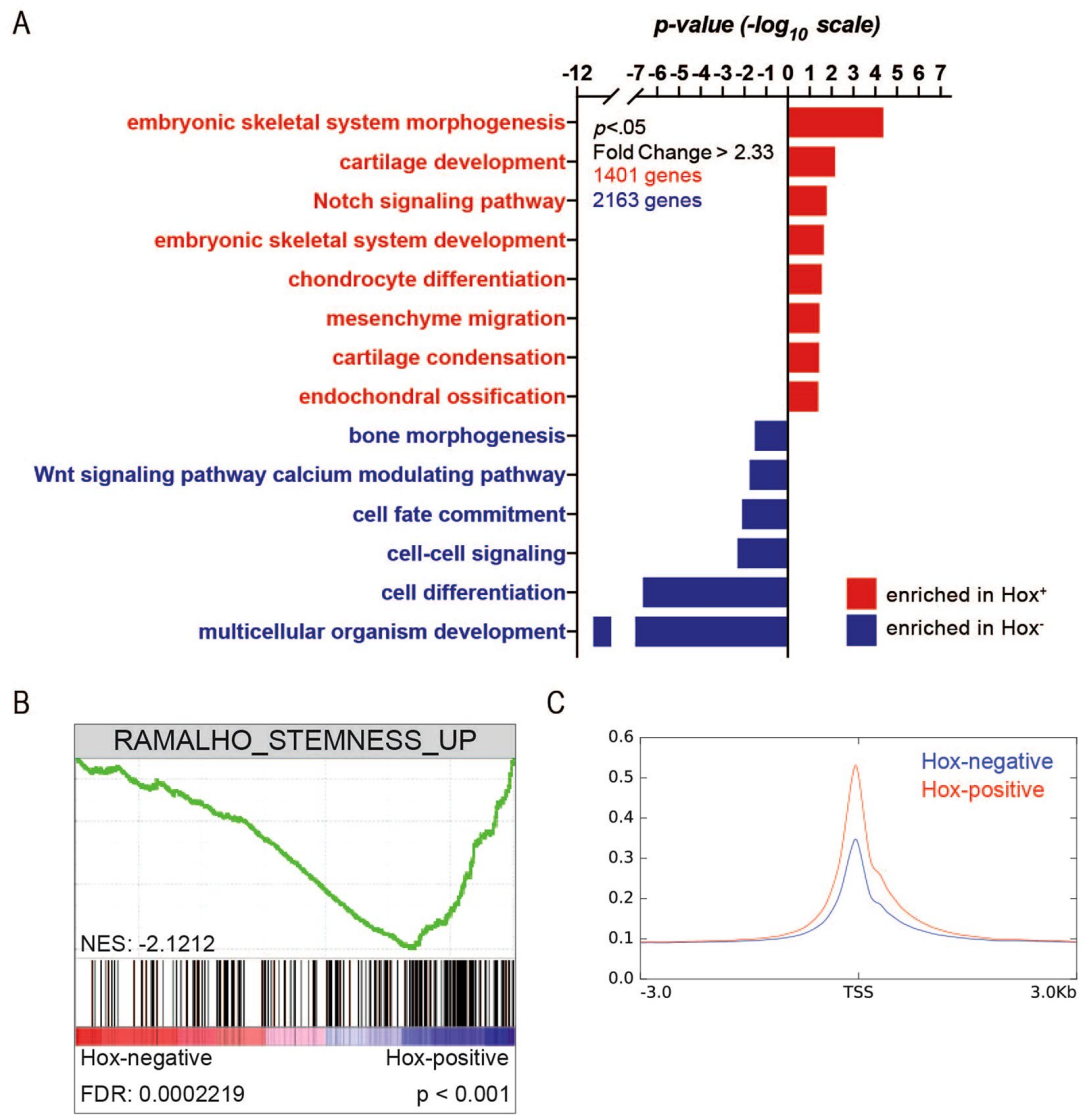
Gene ontology analysis of *Hox*^+^ vs. *Hox*^***−***^ periosteal cells. (**A**) *Hox*^+^ periosteal cells are enriched in biological processes associated with stem-like cells, such as embryonic skeletal system morphogenesis and development, and mesenchyme migration (red), while the *Hox*^***−***^ periosteal cells exhibit features characteristic of more committed progenitor cells and terminally differentiated cells (blue). GO categories ranked by significance (*p* ≤ 0.05 or −log_10_(*p*) ≥ 1.3). (**B**) Gene set enrichment analysis (GSEA) plots demonstrate that *Hox*-positive SSCs positively correlate with the gene set for stemness (RAMALHO_STEMNESS_UP). (**C**) Aggregated enrichment of ATAC-seq signal around all transcription start sites in *Hox*^+^ (red) and *Hox*^*-*^ (blue) periosteal cells.

These data suggest that *Hox* gene expression imparts tri-lineage potential on the periosteal stem/progenitor cell, while loss/suppression of *Hox* expression leads to progression on the lineage tree towards a more committed osteochondroprogenitor cell, osteoprogenitor cell, or osteoblast. We returned to the RNAseq data to confirm this observation. In particular, we employed a gene ontology (GO) analysis to determine the distinct biological functions assigned to either *Hox*^+^ or *Hox*^***−***^ skeletal cells. Gene ontology categories that were enriched in each group revealed that *Hox*^+^ cells share common GO categories such as “embryonic skeletal system morphogenesis”, “embryonic skeletal system development”, and “mesenchyme migration”, suggesting a less committed, more primitive stem-like cell population (Fig. 7A). This is in contrast to *Hox*^***−***^ cells, which show enrichment in GO categories such as “bone morphogenesis”, “cell fate commitment”, and “cell differentiation”, indicating a more committed progenitor population (Fig. 7A). Gene set enrichment analysis demonstrates biological processes modulated by *Hox* gene expression. GSEA analysis of GO terms revealed that *Hox* gene expression regulates gene sets associated with stemness (Fig. 7B). Finally, we utilized ATACseq to quantify the accessibility of chromatin in *Hox*-positive and *Hox*-negative SSCs. Increased accessibility of chromatin is associated with stemness^23^, and our analysis revealed that *Hox*-positive periosteal stem/progenitor cells exhibited a larger area of open chromatin near transcriptional start sites compared to *Hox*-negative periosteal stem/progenitor cells (Fig. 7C).

Taken together, the current study provides the first experimental evidence that *Hox* gene expression in adult periosteal stem/progenitor cells imparts lifelong genetic regulation of tri-lineage differentiation potential of these cells. Notably, we provide proof that periosteal stem/progenitor cells are not just one cell population, equal in function and character, but rather a diverse population with distinct differentiation potential, likely responsible for the unique regenerative response seen in different skeletal elements.

## Discussion

In comparison to other tetrapod vertebrates, humans possess limited regenerative capacity. While amphibians can replace entire limb segments^24,25^ including the most complex musculoskeletal structures – mammals, including humans, can only partially regenerate injured bone segments, let alone restore entire appendages. Previous work has identified maintenance of *Hox* gene expression in adult mouse skeletal stem cells^4,26^. *Picchi et al*. and *Ackema et al*. have both reported the presence of a Hox code in bone marrow-derived stromal cells, consistent with the embryonic Hox pattern, and both have suggested a role in determining cellular identity and function in adulthood^6,7^, but no studies have yet identified a functional role of *Hox* gene expression in adult skeletal stem/progenitor cells specifically in the periosteum. The current study presents first evidence that *Hox* gene expression provides periosteal skeletal stem/progenitor cells with an anatomic signature, and at the same time imparts differentiation cues to these stem cells, which is a prerequisite for successful skeletal element regeneration.

Our previous work has shown that *Hox* gene expression of adult tibial skeletal stem cells confers upon them a sense of positional identity, which is unchanged when cells are placed into a new environment^4^, similar to experiments performed on embryonic tissues^27^. While this positional memory of the skeletal stem cell may be important in transplantation experiments and surgical procedures, it is not clear why skeletal stem/progenitor cells possess and maintain this identity into adulthood. Should we consider bone regeneration that occurs after fracture in a human or mouse as complex as limb regeneration in an axolotl after amputation – one that requires positional identity to undergo proper morphogenesis of specific bone or limb segments? What if organization of the early hematoma after fracture requires a “Bauplan” similar to the one established by the *Hox* expression patterning during embryonic development or in a blastema after amputation? *Rux et al*. recently published compelling data that Hox expression is limited to adult progenitor-enriched mesenchymal stem/stromal cells and is essential for proper differentiation during repair^26^. In response to injury, it is this cell population that is essential for successful regeneration^28^, but their initial prevalence is miniscule compared to the other cell types present in the hematoma. Communication between these few cells is likely impossible due to the physical distance between them, therefore each skeletal stem cell has to be equipped with an architectural plan outlining the final regenerative product. Similar to the developing embryo, the establishment of cellular and tissue compartments within an early regenerate occurs in an environment, where gradients of transcription factors cannot be established over many cell diameters, nor can extracellular signaling proteins/morphogens modulate positional identity of this scarce subset of cells^29,30^. Activation and maintenance of *Hox* gene expression in the skeletal stem cell pool is auto- and cross-regulated by products of *Hox* genes themselves, as well as through a group of proteins that modulate transcription and chromatin conformation, the Polycomb and Trithorax group. lncRNAs, such as HOTTIP and HOTAIRM1 can bind to these complexes and can thus modulate Hox gene expression^31^. We took advantage of this regulatory mechanism and manipulated Hox gene expression using siRNA and antisense oligonucleotides against these lncRNAs. Using this strategy, we gathered crucial information on the functional role of *Hox* gene expression in adult periosteal stem/progenitor cells. First, our *in vitro* and RNAseq data provide convincing data that the presence or absence of *Hox* expression delineates two very distinct populations of periosteal stem/progenitor cells, varying not only in a vast number of transcriptional products, but also demonstrating unique cell fate decisions. While *Hox*-negative periosteal stem/progenitor cells favor osteogenesis, *Hox*-positive periosteal stem/progenitor cells primarily differentiate into cartilage and fat. When *Hox* expression was suppressed using siRNA or ASOs, we observed a reduction of the pro-chondrogenic and pro-adipogenic phenotype in the *Hox*-positive periosteal stem/progenitor cells population towards a more osteogenic phenotype. While these data only provide evidence that Hox paralogs impart differential functional information on periosteal stem/progenitor cells when *Hox* genes are ON or OFF, it remains unknown whether different *Hox* genes convey specific and unique patterning, repair and morphology instructions to the regenerating skeletal element. This may add critical complexity to the scientific journey towards mammalian limb regeneration and may revolutionize the procedure of bone grafting in orthopaedic surgery and dentistry. If the positional identity of a skeletal stem cell is essential for its regenerative capacity, then bone grafting procedures should take this positional information into account when the graft donor site is chosen.

## Materials and Methods

### Mice

All procedures were approved by the New York University Committee on Animal Research and were performed in accordance with the institutional guidelines and regulations. The studies were conducted on 12-week-old C57BL/6J male mice purchased from Jackson Laboratories (Bar Harbor, ME).

### Periosteum dissection and RNA isolation

The periosteum from four different skeletal sites (F-frontal bone, H-hyoid bone, P-parietal bone, T-tibia), each representing a unique signature of embryonic Hox code (positive/negative) and embryonic origin (neural crest (NC)/mesoderm) were analyzed. After the soft tissues were carefully removed, the periosteum was collected with a Gracey curette (Hu Friedy, Chicago, IL) and stored in RNAlater (Qiagen, Hilden, Germany). The tissues were homogenized in Qiazol (Qiagen) with a mortar and pestle complemented with Qiashredder columns (Qiagen) then purified with the miRNeasy kit (Qiagen) following the manufacturer’s instructions. The RNA purity was verified with Nanodrop (Thermo Fisher Scientific, Waltham, MA) and the integrity was evaluated with Bioanalyzer (Agilent Technologies, Santa Clara, CA). Only samples with 260/230 ratios superior to 2.0 and RIN superior to 8 were further analyzed.

### RNA sequencing

RNA sequencing was performed utilizing the high output, paired-end reads with the Illumina HiSeq. 2500 System. Bioinformatic analyses were performed with the Tophat (version 2.0.9) alignment program for reads mapping with two mismatches allowed. Cufflinks (version 2.2.0) was used to calculate FPKM values, and Htseq (version 0.6.1.p.1) was used to find the read counts for annotated genomic features. For the differential gene statistical analysis, DESeq. 2 R/Bioconductor package in the R statistical programming environment was used. The contrast groups for the comparison were: Frontal (F) (2 replicates) versus hyoid (H) (2 replicates), parietal (P) (2 replicates) versus tibia (T) (2 replicates), neural crest (ND) (F + H, 4 replicates) versus mesoderm (MD) (P + T, 4 replicates) and Hox-negative (F + P, 4 replicates) versus Hox-positive (H + T, 4 replicates). Gene set enrichment analysis was performed using the GSEA software (http://www.broadinstitute.org/gsea/index.jsp) on log2 expression data of periosteal cells from the four bones aforementioned and classified in the corresponding classes. Gene sets were taken from the Molecular Signatures Database (http://www.broadinstitute.org/gsea/msigdb/index.jsp). In particular, we investigated whether periosteum from each source was associated with over- or under-represented genes in pairwise comparisons between each class and the rest. Gene sets were permuted 1000 times, the normalized enrichment score (NES) was calculated for each gene set, the nominal P value was obtained and the false discovery rate (FDR) was calculated to correct the P value.

Gene ontology (GO) analysis was performed using the Database for Annotation, Visualization, and Integrated Discovery (DAVID; http://david.abcc.ncifcrf.gov/). Genes that were significantly enriched (*p* ≤ 0.05; ≥2.33-fold) in *Hox*^+^ vs. *Hox*^***−***^ periosteal cells (1401 genes) or *Hox*^***−***^ vs. *Hox*^+^ periosteal cells (2163 genes) were used to generate GO terms corresponding to biological processes (with GOTERM_BP_DIRECT for functional annotation). Those significantly associated (*p* ≤ 0.05 or −log_10_(*p*) ≥ 1.3) with the gene list are ranked by *p*-value.

### ATACseq analysis

ATACseq differential chromatin detection analysis was performed for three lanes of a paired-end 50 Illumina HiSeq. 2500 run. Per-read per-sample FASTQ files were generated using the bcl2fastq Conversion software (v1.8.4) to convert per-cycle BCL base call files outputted by the sequencing instrument into the FASTQ format. The alignment program, Bowtie2 (v2.3.4.1), was used for mapping reads of 18 mouse samples to the mouse reference genome mm10 and the application Sambamba (v0.6.7) was utilized to remove duplicate reads. The algorithm, MACS (in Python v2.7.3), was used to call peaks of signal for annotated genomic features and, similarly, the Python package NucleoATAC was functioned to call nucleosome positions. The computeMatrix and plotProfile tools in the deepTools suite (v2.3.3) were utilized for generation of signal profile plots. For the differential peak statistical comparisons between six groups of samples with three replicates each, the DiffBind package (Bioconductor v3.3.0) in the R statistical programming environment was utilized. Venn diagrams of differentially detected annotated genomic features comparing the ATACseq sample datasets with the corresponding sample sets from a related RNAseq experiment were generated using the Venny web application (v2.1).

### Flow cytometry

Tibial, hyoid, frontal, parietal bones were harvested and periosteal cells were isolated as previously described. Dissociated cell samples were stained with PE-conjugated antibodies against CD31, CD45, and Ter-119 (Miltenyi Biotec) and APC-conjugated antibodies against Sca1, CD146, or CD166 (Miltenyi Biotec) for purification by flow cytometry (Beckman-Coulter Moflo XDP, Brea, CA). CD31−, CD45−, Ter-119−, and Sca1+, CD146+, or CD166+ cells were identified separately as distinct populations of skeletal stem/progenitor cells.

### Quantitative RT-PCR

RNA was isolated from frontal, parietal, hyoid bones and the tibia as described and then reverse transcribed into cDNA with the Omniscript RT kit (Qiagen). The cDNA was amplified for specific targets using specific primers (listed in Table 1) and RT2 SYBR Green ROX PCR Master Mix in a QuantStudio3 Real-Time PCR System (Thermo Fisher Scientific). Results are presented as 2^−ΔΔCt^ values normalized to the expression of 18S or beta-actin and negative control samples. All reactions were performed in triplicate; means and standard deviations were calculated in GraphPad Prism 7 software.

**Table 1:** PCR primers. All primers were purchased from Integrated DNA Technologies.

Primer Name	Sequence (5′-3′)
*18S* FOR	ACGAGACTCTGGCATGCTAACTAGT
*18S* REV	CGCCACTTGTCCCTCTAAGAA
*Beta-Actin* FOR	TGTTACCAACTGGGACGACA
*Beta-Actin* REV	CTGGGTCATCTTTTCACGGT
*Hoxa2* FOR	GTCGAGGTCTTGATTGATGAACT
*Hoxa2* REV	GTCGAGGTCTTGATTGATGAACT
*Hoxa11* FOR	CTCCAGCCTCCCTTCTTTTT
*Hoxa11* REV	AGTAGCAGTGGGCCAGATTG
*Hoxa13* FOR	CTGGAACGGCCAAATGTACT
*Hoxa13* REV	CCTCCGTTTGTCCTTGGTAA
*Hotairm1* FOR	AATCGGGGCAACTCTGCTAC
*Hotairm1* REV	AGCATGCTCCTGGGTCTCTA
*Hottip* FOR	TCCCGCTTTGTACAGGGAAC
*Hottip* REV	GAGGGGCTTGCTACACCTTT
*Osterix* FOR	GGAGACCTTGCTCGTAGATTTC
*Osterix* REV	GGGATCTTAGTGACTGCCTAAC
*Type I Collagen* FOR	CAGTCGATTCACCTACAGCACG
*Type I Collagen* REV	GGGATGGAGGGAGTTTACACG
*Sox9* FOR	TACGACTGGACGCTGGTGC
*Sox9* REV	TTCATGGGTCGCTTGACGT
*Type II Collagen* FOR	TCCAGATGACTTTCCTCCGTCTA
*Type II Collagen* REV	CAGGTAGGCGATGCTGTTCTTAC
*Papr-γ* FOR	ATAGGTGTGATCTTAACTGCCG
*Ppar-γ* REV	CCAACAGCTTCTCCTTCTCG
*Fabp4* FOR	AAGAAGTGGGAGTGGGCTTT
*Fabp4* REV	AATCCCCATTTACGCTGATG

### Periosteal injury

In order to compare the periosteal reaction of upon injury amongst the different bones, we surgically performed a periosteal scratch on the frontal, parietal, hyoid bones and the tibia. After anesthesia was induced with Isoflurane inhalation (1–5%), 3 mm incisions through the skin were created on the head, neck and shin then the periosteum from the aforementioned bones was scratched with the tip of a 27-gauge syringe needle. The skin was closed and the bones were allowed to heal for 7 days. Non-injured bones were used as control.

### Histology and immunofluorescence antibody staining

Frontal, parietal, hyoid bones and the tibia with and without periosteal injury were harvested and fixed in 4% paraformaldehyde (n = 5). For bright field microscopy images, the samples were decalcified in 19% ethylenediaminetetraacetic acid (EDTA) for 3 weeks, paraffin-embedded and 10-μm thick sections were stained with Movat’s pentachrome^32^. The sections were examined and photographed using a Leica digital imaging system. For immunofluorescence, samples were decalcified in 19% EDTA for 48 hours then, after cryoprotection in 30% sucrose, embedded in Tissue-Tek OCT media (Sakura Finetek, Torrance, CA) and cryosectioned at 100 μm thickness. Sections were incubated overnight at 4 °C in OSX antibody (rabbit anti-mouse A-13, 1:100, Santa Cruz, Santa Cruz, CA) and SOX9 antibody (goat anti-human, 1:20, R&D). Next, sections were stained with DAPI (Thermo Fisher Scientific), coverslipped with Fluoromount (Thermo Fisher Scientific), examined and photographed using a Zeiss LSM 710 laser scanning confocal microscope.

### *In situ* hybridization

*In situ* hybridization was performed using the RNAscope Probe-Mm-Hoxa2, RNAscope Probe-Mm-Hoxa11 and made-to-order RNAscope Probe-Mm-Hoxa13 (Advanced Cell Diagnostics, Newark, CA). Positive (cyclophylin B) and negative antisense controls were performed on additional samples. Hybridized probes were detected using a manual singleplex RNAscope 2.0 HD brown kit (Advanced Cell Diagnostics) following the manufacturer’s instructions. The sections were examined and photographed using a Leica digital imaging system.

### Periosteal cell isolation

Primary SSCs were obtained from frontal, parietal, hyoid bones and the tibia. After careful dissection, bones with intact periosteum were submitted to 4 serial collagenase digestions in 0.2% collagenase type 2 (Thermo Fisher Scientific) in DMEM at 37 °C for 20 minutes with gentle rocking. After each of the first three digestions, bones were subjected to light centrifugation (1000 rpm) for 5 min and then transferred to a fresh tube of collagenase. After the last digestion, bones were centrifuged at 1400 rpm for 5 min and the pelleted cells were resuspended in growth media. Selective isolation of periosteal stem/progenitor cells was confirmed using FACS analysis.

### *In vitro* differentiation assays

Periosteal progenitor cells from frontal, parietal, hyoid and tibia were isolated as described above and submitted to tri-lineage differentiation. For osteogenic differentiation, cells were cultured in DMEM containing 10% FBS, 100 μg/ml ascorbic acid 10 mM ß-glycerophosphate and 1% penicillin/streptomycin, which was replaced every 3 days. After 21 days, cultures were stained with alizarin red and *in vitro* mineralization was quantified as previously described^33^. For chondrogenic differentiation, micro masses were cultured in chondrogenic differentiation media, supplemented with TGF β-3 (Lonza, Basel, Switzerland). After 14 days, the micro masses were fixed in 4% PFA, photographed under polarized light microscope (Leica) and paraffin embedded. Ten μm-thick sections were obtained and stained with alcian blue and hematoxylin. For adipogenic differentiation, SSCs were cultured in adipogenic differentiation media (Lonza). After 14 days, cells were stained with Oil Red-O and the number of positive cells per well was counted by an examiner blinded to the groups.

### RNA interference

Primary hyoid SSCs were transfected with commercially available Lincode Mouse Hotairm1 SMARTpool siRNAs targeting Hotairm1 with the target sequence UGGUUUACAUGACUAA (GE Dharmacon, Lafayette, CO) and primary tibial SSCs were transfected with Hottip antisense oligonucleotides (ASOs). The sequence for the Hottip ASOs was TAGTGCTTCTAAAACG. A non-targeting ASO control with the sequence AACACGTCATATACGC was used. RNAi Max lipofectamine (Thermo Fisher Scientific) was used as transfection reagent as per manufacturer’s instructions. 24 hours after the transfection, the transfection media was replaced by regular growth media, osteogenic, chondrogenic or adipogenic differentiation media as described. Total RNA was harvested 48 h later using RNeasy Mini Kit (Qiagen). The knockdown of Hotairm1 in the hyoid SSCs, Hottip in tibial SSCs, as well as the downstream effect on the expression of specific Hox genes and tri-lineage differentiation markers were assessed by qPCR as described using specific primers (listed in Table 1).

### Statistical analysis

A priori power analysis to obtain statistical significance (p = 0.05, power 80%) resulted in an n of 5 for each group, expecting a 25% difference between the two groups. Prism 7 (GraphPad Software, Inc., La Jolla, CA) was used for statistical computations. A Student’s *t* test was used for all comparisons in which there were two groups; ANOVA analyses followed by the Holms-Sidak correction for post-hoc testing was applied for analyses in which there were two or more comparisons being made. Error bars represent standard deviation. An asterisk symbol (*) denotes a *p* value of less than 0.05.

## Data Availability

The data that support the findings of this study are available from the corresponding author upon request.
