# Author Correction: Hox gene expression determines cell fate of adult periosteal stem/progenitor cells

**DOI:** 10.1038/s41598-020-59764-z

**Published:** 2020-02-18

**Authors:** Vivian Bradaschia-Correa, Kevin Leclerc, Anne M. Josephson, Sooyeon Lee, Laura Palma, Hannah P. Litwa, Shane S. Neibart, Jason C. Huo, Philipp Leucht

**Affiliations:** 1 Department of Orthopaedic Surgery, New York University School of Medicine, New York, NY USA; 2 Department of Cell Biology, New York University School of Medicine, New York, NY USA

Correction to: *Scientific Reports* 10.1038/s41598-019-41639-7, published online 25 March 2019

In Figure 5, the x-axes have been incorrectly labelled. The correct Figure 5 appears below as Figure 1.

**Figure 1:**
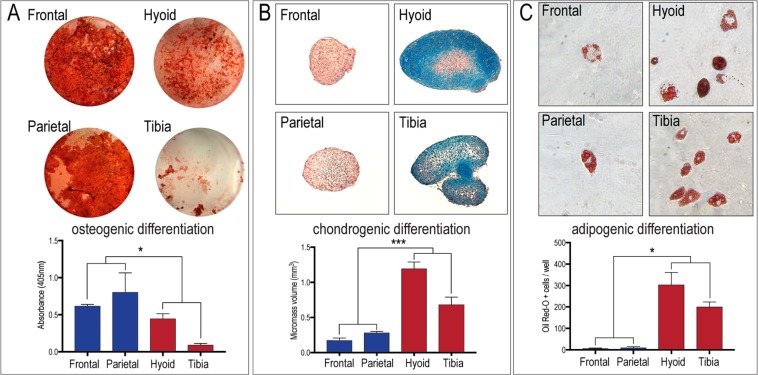
.

